# Editorial: The defense responses of aquatic animals to the environment

**DOI:** 10.3389/fphys.2023.1231014

**Published:** 2023-06-08

**Authors:** José Ricardo Paula, Xiaodan Wang, Chang Xu, Daniel C. Moreira

**Affiliations:** ^1^ MARE—Marine and Environmental Sciences Centre and ARNET—Aquatic Research Network, Laboratório Marítimo da Guia, Faculdade de Ciências, Universidade de Lisboa, Cascais, Portugal; ^2^ Departamento de Biologia Animal, Faculdade de Ciências, Universidade de Lisboa, Lisboa, Portugal; ^3^ Hawaiʻi Institute of Marine Biology, University of Hawai’i at Mānoa, Kaneohe, HI, United States; ^4^ Laboratory of Aquaculture Nutrition and Environmental Health, School of Life Sciences, East China Normal University, Shanghai, China; ^5^ Department of Aquaculture, College of Marine Sciences, Hainan University, Haikou, Hainan, China; ^6^ Research Center in Morphology and Applied Immunology, Faculty of Medicine, University of Brasília, Brasília, Brazil

**Keywords:** environmental stressor, hydrogen sulfide, osmoregulation, non-essential element, pH variation, ultraviolet radiation

Aquatic animals use different physiological processes to maintain balance in response to environmental stressors. This Research Topic gathers innovative research studies that examine animal strategies for coping with various stressors. The studies focus on stressors like ultraviolet radiation (Gu et al.), hydrogen sulfide contamination (Alipio et al.), exposure to non-essential elements (Campoy-Diaz et al.), pH and ion variations (Lin et al.), and low-salinity stress (Li et al.). Additionally, a sixth manuscript published in this Research Topic explores the role of histone acetylation in the inflammatory response during foreign material implantation in pearl production (Yang et al.).

A study conducted by Gu et al. explored the differences in ultraviolet radiation (UVR) tolerance between low- and high-altitude fish species. The researchers analyzed the skin histology of 22 species and observed histological changes after UVR exposure. The study revealed that fish living in high-altitude and cold-water environments have stronger UVR tolerance, suggesting that adapting to such environments may be essential for UVR resistance. This research enriches our knowledge of the influence of environmental factors on UVR tolerance in aquatic animals.

Alipio et al. investigated the impact of hydrogen sulfide (H_2_S) gas on the mucosa of Atlantic salmon (*Salmo salar*), which is known to affect mucosal functions in mammals. Using explant models, the authors focused on genes responsible for mucins and sulfide detoxification. The study results showed that exposure to a sulfide donor impacted the expression of these genes, emphasizing the role of mucins in protecting mucosa against H_2_S toxicity. This research provides valuable insights into the unclear interactions between H_2_S and fish at the mucosal level.

Campoy-Diaz et al. studied the balance between free radical production, antioxidant defenses, and oxidative damage in the digestive gland of the freshwater gastropod *Pomacea canaliculata* after exposure to non-essential elements (mercury, arsenic, and uranium). The authors found that snails exhibited increased levels of reactive species and oxidative damage, which were partially compensated by non-enzymatic antioxidant defenses. This work sheds light on the cellular mechanisms underlying this gastropod’s tolerance to non-essential elements and supports its use as a bioindicator species for metal pollution in freshwater bodies.

Using a transcriptomic approach, Li et al. investigated the molecular mechanisms underlying the tolerance of the black tiger shrimp, *Penaeus monodon*, to low-salinity stress. Chronic exposure to low salinity triggered adjustments in several cellular processes in the gills of these commercially relevant shrimps. There was a salinity-dependent response of differentially expressed genes, in which the major affected pathways were metabolic, immune and signaling pathways. Overall, the study revealed key molecular events associated with the survival of *P. monodon* to long-term low salinity stress. These insights are useful in understanding animal adaptation to environmental changes in salinity in nature or in aquaculture settings.

Lin et al. investigated the role of vitamin D in the regulation of ionocyte differentiation in zebrafish (*Danio rerio*) larvae. 1α,25-dihydroxyvitamin D3 (1α,25(OH)2D3), the bioactive form of vitamin D, is a steroid hormone that is involved in the regulation of Ca2+ uptake and acid secretion in teleosts. The present study indicated that 1α,25(OH)2D3 treatment increased the number of foxi3a-positive cells, ionocyte progenitors, and mature ionocytes. However, the number of P63-positive cells and epidermal stem cells did not change in the zebrafish larvae treated with 1α,25(OH)2D3. This study indicated that vitamin might play a positive effect on the ionocyte differentiation, which would increase the ion regulation ability of freshwater teleosts. The findings in this paper would provide some new insights into the mechanisms of how fish cope with environmental stress.

Finally, Yang et al. also used a transcriptomic approach to understand the role of histone acetylation in the modulation of the inflammatory response of the pearl oyster *Pinctada fucata*. The immune response of oysters is a decisive factor for the success of xenograft mantle transplantation, a technique used for pearl production. After successfully inducing H3 acetylation (butyrate treatment), the authors identified differentially expressed genes involved in the lysosomal pathway (upregulated) and cell migration and cell proliferation (downregulated). This effect was coupled with increased levels of lipid peroxidation and activity of antioxidant enzymes. In sum, the study revealed that histone H3 acetylation effectively alters the immune response of oysters to transplantation and identified the underlying molecular mechanisms. This information might support the improvement of the health and immunity of pearl oysters and ultimately optimize pearl production.

In conclusion, the studies in this Research Topic provide valuable insights into the defense mechanisms employed by aquatic animals in response to various environmental stressors. Understanding these defense responses is essential for predicting risks, determining future conservation strategies, and informing environmental protection programs.

